# Intrahepatic arterial localizer guided transjugular intrahepatic portosystemic shunt placement: Feasibility, efficacy, and technical success assessed by a case series-a STROBE- compliant article

**DOI:** 10.1097/MD.0000000000016868

**Published:** 2019-08-16

**Authors:** Wang Haochen, Zou Yinghua, Wang Jian

**Affiliations:** Department of Interventional Radiology and Vascular Surgery of Peking university first hospital, Beijing, China.

**Keywords:** cone beam CT, hepatic artery, liver cirrhosis, portosystemic shunt

## Abstract

Transjugular intra-hepatic portosystemic shunts (TIPS) had been considered a standard procedure in patients suffering from portal hypertension. The most challenging step in TIPS placement is blind puncture of the portal vein. We had established a localization method by introducing an Intra-Hepatic Arterial based puncture directing Localizer (IHAL) with the assistance of the enhanced computed tomography (CT) reconstruction. This study aimed to evaluate the feasibility, efficacy, and technical success of this method.

From June 2018 to August 2018, 10 consecutive patients suffering from refractory ascites or esophageal gastric bleeding by liver cirrhosis were included in this retrospective study to evaluate feasibility, efficacy, and technical success of enhanced CT assisted IHAL-guided puncture of the portal vein. As a control, 10 patients receiving TIPS placement before Jun 2018 with cone beam CT (CBCT)-guided puncture were included to compare the reduction of portal-systemic pressure gradient (PSPG), portal entry time (PET), the number of puncture, dose area product (DAP) and contrast medium consumption.

Technical success was 100% in the study group (IHAL-guided group) and in 90.0% of the control group (CBCT-guided group). Appropriate IHAL point could be achieved in all patients under the enhanced CT reconstruction assistance. The median number of punctures and DAP in IHAL group were significantly less than those in CBCT group. The reduction of PSPG, PET, and contrast medium consumption in IHAL group showed no significant differences than those in CBCT group.

Enhanced CT reconstruction assisted IHAL-guided portal vein puncture is technically feasible and a reliable tool for TIPS placement resulting in a significant reduction of the number of punctures and DAP.

## 1 Introduction

Transjugular intrahepatic portosystemic shunt (TIPS) is established as an effective, durable therapy for treating complications of portal hypertension.^[1,2,3]^ However, TIPS placement remains a challenging procedure with a risk of technical failure and complications arising from portal vein puncture attempts, which can result in abdominal bleeding, hemobilia, transcapsular puncture, and hepatic artery injury.^[4]^ The puncture of portal vein can be guided by a 2-dimensional portal image by wedged portography using carbon dioxide or an iodinated contrast medium as well as trans-splenic and transarterial mesenteric indirect portography.^[4,5]^ Cone-beam computed tomography (CBCT) with image fusion techniques have been designed to gain portal vein access with a higher successful rate.^[6]^ Other guiding techniques including percutaneous balloon insertion or ultrasound-guided puncture have been described before.^[4,7,8,9,10,11]^ In our institution, we designed a portal localization method with an intra-hepatic arterial localizer (IHAL) under enhanced CT reconstruction assistance. In this article, we summarized the feasibility, efficacy, and technical success of this puncture guiding method for TIPS placement.

## 2 Methods

### 2.1 Study population and control group

This study was approved by the Ethics Committee of Peking University First Hospital. Informed consent for the TIPS placement was obtained from all individual participants included in the study. From June 2018 to August 2018, A total of 10 consecutive patients (median age 61 years, range 43–82 years with liver cirrhosis suffering from refractory ascites (n = 8) or esophagogastric varices (n = 2) undergoing IHAL-guided TIPS placement were included in this study. Underlying causes in development of liver cirrhosis and subsequently portal hypertension were hepatitis B-virus infection (n = 4), hepatitis C-virus infection (n = 4), alcoholic hepatitis (n = 2). Refractory ascites was defined according to the CIRSE guidelines as an abdominal fluid collection that cannot be mobilized or the early recurrence of abdominal fluid that cannot adequately be prevented by medical therapy.^[12]^ As a control group to make the comparison, 10 patients (median age 62 years, range 45–79 years) with indication for TIPS placement of refractory ascites (n = 7) or esophagogastric varices (n = 3) undergoing TIPS placement by the guidance of CBCT before Jun 2018 were also included in this study. Patient characteristics are summarized in Table 1.

**Table 1:**
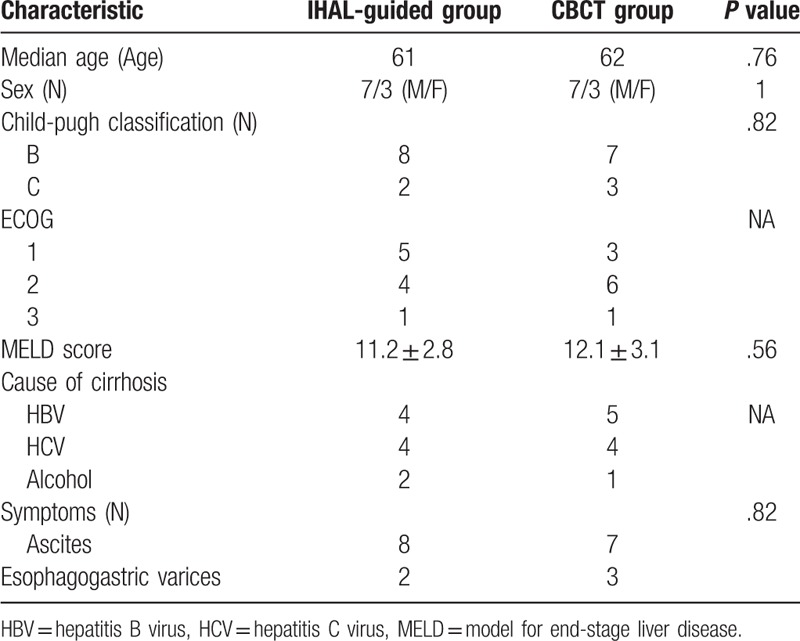
Baseline in the 2 groups.

### 2.2 Determination of the IHAL location on enhanced CT reconstruction image

**Figure 1:**
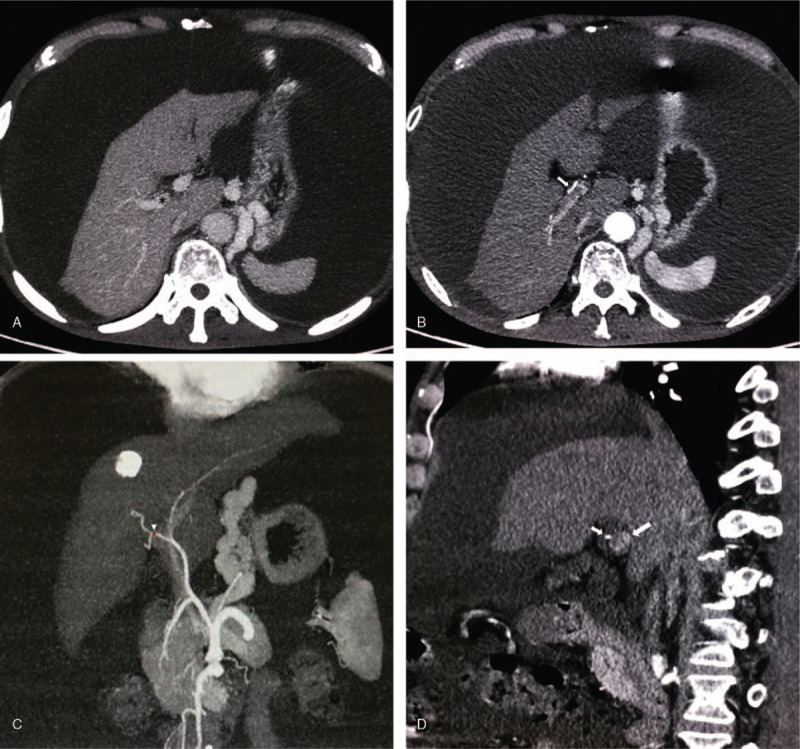
A. On the portal phase of the CT, the target entry zone of portal vein (∗) was determined. B. IHAL point (white arrow) ventral to the scheduled entry zone of the portal vein. C. A MIP image showed hepatic artery branch with the localization point on it (white arrow head). D. A MIP image showed the IHAL point (small arrow) and entry area of the portal vein (big arrow). IHAL = intrahepatic arterial localizer, MIP = maximum intensity projection.

The location of the IHAL was determined in advance on the multi-phase enhancement CT scan which was obtained within 1 week before the TIPS placement. The process was summarized as follows: on the portal phase of the enhanced CT ultrathin sliced image, we determined the entry point of portal vein on an axial image (Fig. 1A), and then, we switched to arterial phase of the same slice, and moved the mouse courser ventrally to reach the arterial point perpendicular to the scheduled entry point of the portal vein (Fig. 1B). Then we switched to a coronal maximum-intensity-projection (MIP) image and adjusted the window width, till the whole projection of the hepatic artery branch can be visualized with the localization point on it (Fig. 1C). This point was the site where the IHAL should be placed. When the localization point was determined, the puncture route was easily scheduled on antero-posterior projection and lateral projection with portal phase MIP CT reconstruction image (Fig. 1D).

### 2.3 Placement of the IHAL

**Figure 2:**
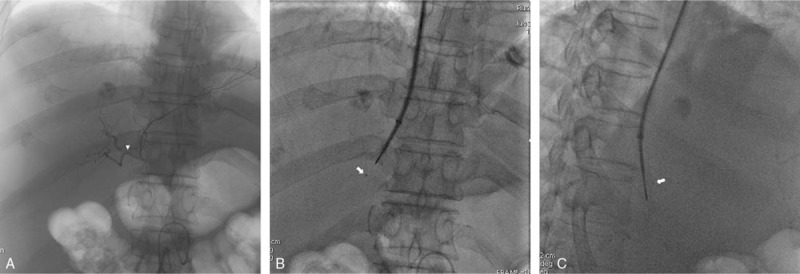
A. IHAL point (arrow head) was shown on an angiography from the common hepatic artery. B. Puncture was done under the guidance of IHAL point (arrow head), a P-A view. C. Puncture was done under the guidance of IHAL point (arrow), a lateral view. IHAL = intrahepatic arterial localizer.

In this study, for the purpose of intravascular use, we chose a super-selective microcatheter with radiopaque marker on the top as the IHAL. Placement of IHAL was done before TIPS placement. Catheterization of the celiac artery was done by a 5F angiographic catheter, after angiography and visualization of the whole hepatic artery (Fig. 2A), a 2.7F micro-catheter (Asahi Intecc Co. Ltd, Japan) was introduced to reach the pre-determined IHAL point according to CT reconstruction images, the radiopaque marker on the top of the micro catheter will serve as the localizer for the following puncture process (Fig. 2B and C). The spatial relationship between the localizer and the entry point of portal vein remained constantly despite of the organ movement when breathing, which made the target for puncture more accurately.

### 2.4 TIPS Procedure

**Figure 3:**
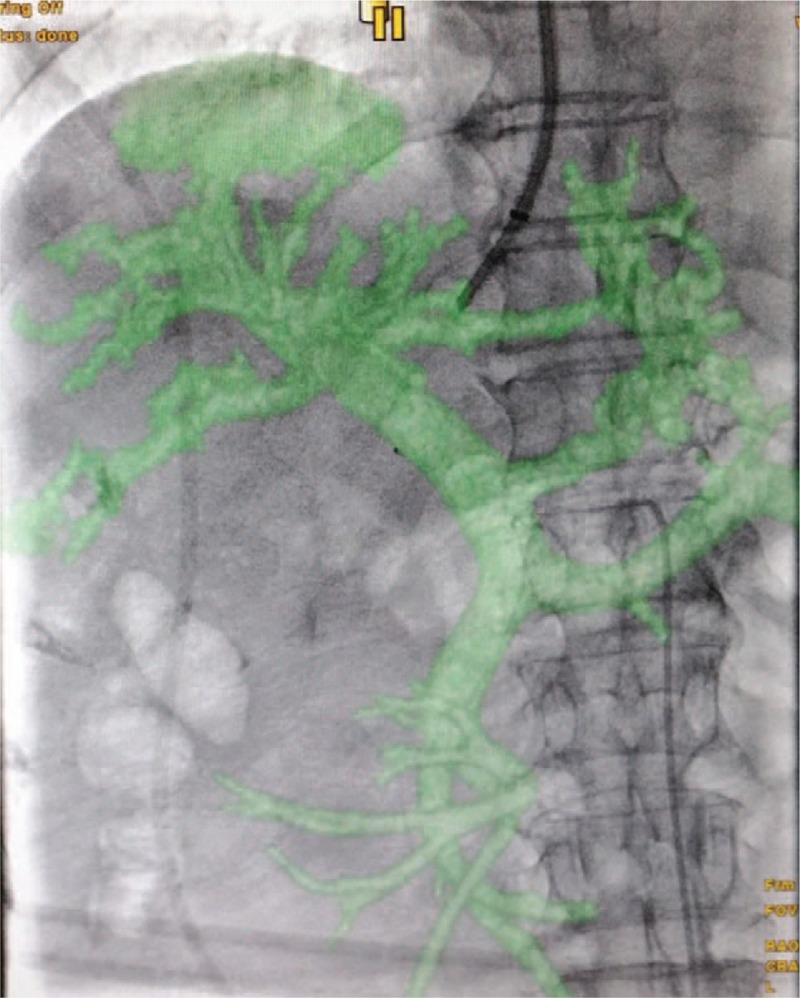
Portal vein puncture was guided under the guidance of a real-time fused imaging technique.

**Figure 4:**
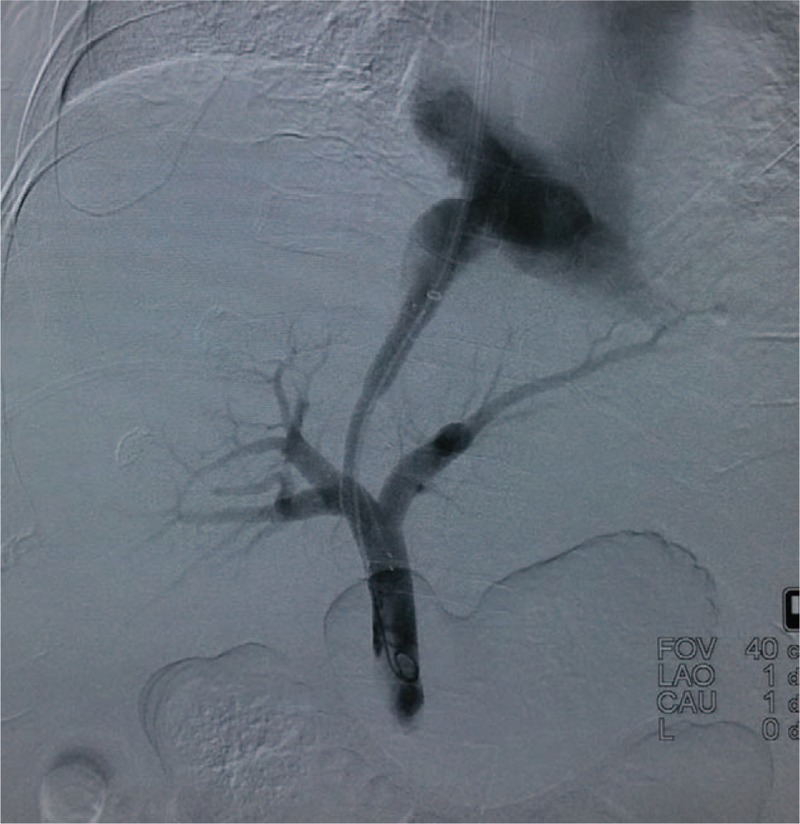
TIPS was successfully placed under the guidance of IHAL (the same patient as Figs. 1 and 2). IHAL = intrahepatic arterial localizer, TIPS = transjugular intra-hepatic portosystemic shunts.

All the procedures were performed under the basal intravenous anesthesia. Angiographic system (GE Medical system, USA) equipped with a flat panel detector was used for all cases. The team performing the procedures included at least 1 senior interventional radiologist with more than 20 years of experiences. For the study group, puncture of the portal vein was performed by the guidance of IHAL. For the control group, CBCT acquisition was performed and then coordinated with the pre-procedural portal phase CT images for image fusion. portal vein puncture was performed under the guidance of the real-time fused imaging technique (Fig. 3). Transjugular needle was passed from the chosen hepatic vein through the liver parenchyma into an intrahepatic branch of the portal vein with a transjugular liver access set (RUPS-100; Cook, Bloomington, Ind). Once the portal vein was punctured and catheterized, the systemic and portal vein pressures were measured directly through the transjugular access. The intrahepatic tract between the hepatic and portal veins was dilated with a 6 × 60 mm balloon, and an e-PTFE-covered stent (8 × 80–100 mm, fluency, bard, USA) was deployed. The stent was then dilated gradually between 6 and 8 mm until satisfactory pressure levels were reached (Fig. 4). Angiographic and hemodynamic assessments of the resulting pressure reduction were then performed. Combined collateral variceal vein embolization was performed if necessary, according to guidelines.^[11]^ By the end of the TIPS placement, angiography of the whole intrahepatic artery was performed to make sure no artery injuries happened during the puncture procedure.

### 2.5 Success assessment

In this study, we compared IHAL guiding method with CBCT guidance in the following aspects:

1. portal entry time (PET), which is from the start of the procedure to the time of an angiographic catheter entering into the main portal vein.
2. The number of punctures.
3. X-ray absorption dosage during PET.
4. Volume of the contrast medium consumption.

Feasibility was defined by the initial technical success of TIPS placement including TIPS patency (between the hepatic vein and a branch of the portal vein at the end of the procedure). Hemodynamic success was defined as a reduction of the portosystemic gradient to a level of 5 to 12 mmHg. Safety data were collected 72 hours after TIPS placement and included the advent of death, abdominal bleeding, hemobilia, capsular puncture, and hepatic artery injury. Effectiveness was defined as the clinical success, that is, resolution of the clinical symptom of hypertension for which the procedure was performed and the absence of major complications related to the TIPS placement.

### 2.6 Statistics

Statistics were performed using the statistical software SPSS 21 (IBM, USA). Median and inter quartile ranges were given for descriptive statistics. The independent *t* test for non-normal distributed data was applied to assess level of significance. For categorical data, *F* test was used. A *P* value lower than .05 was accepted as a significant difference.

## 3 Results

Successful puncture of the portal vein and establishment of a guide wire was achieved in 100% (10/10 patients) of the study group and in 90.0% (9/10patients) of the control group (CBCT-guided group). All procedures with successful puncture of the portal vein and established guidewire resulted in technical success of TIPS placement (reduction of porto-systemic pressure gradient (PSPG) to 10 mm Hg or below) (19/19patients). The porto-systemic gradient in the study and control group were 23.2 ± 4.7 mmHg and 25.9 ± 7.1 mmHg before and 5.5 ± 2.9 mmHg and 7.3 ± 2.3 mmHg after TIPS placement, respectively. No major complications occurred in the study group. In the control group, 1 complication occurred consisting of a transient onset of the bloody ascites after TIPS placement, which suggested puncture of the liver capsule. One patient of the control group failed TIPS placement because of the slim size of the portal vein branch and the breath movements were hardly control which made the CBCT guided portal puncture failed. The median number of punctures in the IHAL-guided study group were 2 ± 1.3 punctures, ranging from 1 to 5 punctures. The median number of punctures in the CBCT-guided control group were 3 ± 2.5 punctures, ranging from 2 to 8 punctures. The PET was 14.8 ± 8.2 min in the study group and 15.0 ± 22.7 min in the control group. The fluoroscopy time was 19.9 ± 12.1 min in the study group and 20.4 ± 17.6 min in the control group. Dose area product (DAP) was 176.6 ± 107.1 Gy × cm^2^ in the study group and 221.1 ± 111.1 Gy × cm^2^ in the control group. Contrast volum was 67.2 ± 12.1 ml in the study group and 64.4 ± 13.4 ml in the control group. All these data were shown in the Table 2.

**Table 2:**
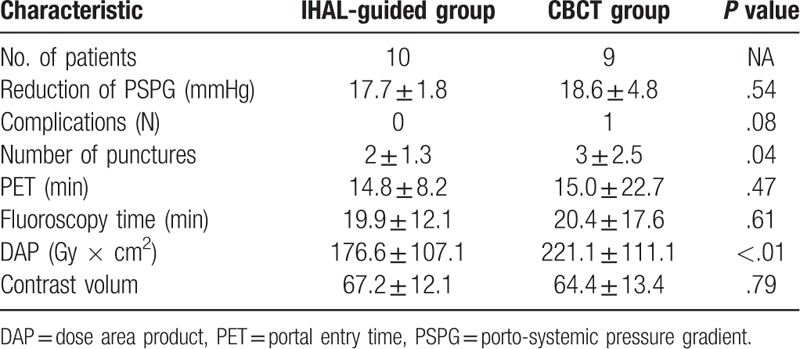
Detailed information of the results data.

## 4 Discussion

With the progress of TIPS placement, various techniques have been described to improve the efficacy of imaging guided portal vein puncture, including transhepatic catheterization of the portal vein, transarterial mesenteric indirect portography,^[13]^ real-time percutaneous or intravasal ultrasound guidance, or wedged portography.^[14,15,16,17]^ Nevertheless, those techniques have their own shortcomings. Real-time percutaneous ultrasound guidance offers a noninvasive method, which has infrequently been widely used. Miraglia et al^[18]^ compared radiation dose from ultrasound-guided TIPS using flat panel detectors and image intensifier systems to fluoroscopic needle guidance for the PV puncture: DAP and fluoroscopy times were lower for ultrasound-guidance versus fluoroscopy. Ultrasound-guided approach is a good method. However, ultrasound operation needs rich experience and cooperation of many people. Intravasal ultrasound-guided intervention requires special equipment and a second clinician familiar with TIPS in some cases resulting in additional costs to the procedure. Transarterial mesenteric indirect portography requires using a large amount of contrast medium which is thought not good for renal function. What's more, sometimes we could not get a clear visualization of portal vein by transarterial mesenteric indirect portography especially on the lateral projection. transhepatic catheterization of the portal vein for guidance is with the additional risk of abdominal bleeding and be used only in some occasions. CBCT guidance for portal puncture has been investigated recently and makes a promising progress in TIPS placement. In our previous procedures with CBCT guided TIPS, we found the breathing movement will affect the results of the coordination of CBCT image with the originate enhanced-CT image, which will affect the accuracy of CBCT-guided portal vein puncture. Although breathing practice has been advocated to the patient, it still difficult to make a strict breath control on some occasions.

The advantages of doing TIPS puncture under IHAL guidance are as follows:

1. Being wrapped in the Glisson sheath, the special relationship between IHAL, and targeting portal entry point is constantly existed, which makes the respiratory movement can be neglected during portal puncture.
2. Indirect portal venography can be omitted which will save a large amount of contrast medium usage that is not good for renal function.
3. With a clear visualization of the IHAL, the target zoon of puncture can be accurately determined both on P-A or lateral projection.
4. IHAL can effectively avoid puncture of the hepatic artery branch.
5. IHAL is clinically available, a selective angiographic microcatheter with a radiopaque distal marker can perfectly serve the purpose.

The study of arterial targeting for TIPS puncture was first introduced by Osamu Matsui et al in 1994,^[19]^ and then further reported by Yamagami et al.^[20]^ In those previous study, the author advocated puncture of the portal vein 1 cm posterior to the targeting guidware placed in celiac trunk which will promote the successful rate of TIPS puncture. In our study, we determined in advance the entry point of the portal side with enhanced CT reconstruction, then we chose the hepatic artery adjacent to the portal entry point for IHAL placement. The distance between IHAL and the entry point of portal vein was different from case to case, this distance could be pre-calculated on the enhanced CT image.

In this study, we found the number of punctures of the 2 groups were statistically different, IHAL -guided procedure had a less puncture number than that of the CBCT group. Also, we found during PET, the X-ray dose of IHAL -guided group was statistically lower than that of the CBCT group. As for the PET, we found no statistically difference in the 2 groups. The reason for that was: with IHAL guidance, we still needed to repeatedly reshape the RUPS-100 system to make sure the angle of its tip adapts to the puncture route. We did not choose the whole procedure time for comparison, because the following embolization of the collaterals after entry of portal vein were quite different among patients.

In conclusion, our study suggests that IHAL-guidance for TIPS placement is feasible and effective. This method reduces the number of puncture and the radiation exposure for the patients. Further studies with a larger patient population should now be conducted to confirm the future usage of this guiding method.

Limitations of the study: anatomical variations of the celiac trunk or extremely tortuous intrahepatic artery will add additional difficulties in IHAL placement, more cases should be investigated in such circumstances.

## Acknowledgments

The authors thank professor Tong Xiaoqiang, Song li and Dr. Lv Tianshi for technical assistance.

## Author contributions

**Investigation:** Wang Haochen.

**Software:** Wang Haochen.

**Supervision:** Wang Jian, Zou Yinghua.

**Visualization:** Wang Haochen.

**Writing – original draft:** Wang Haochen.

**Writing – review & editing:** Wang Jian, Zou Yinghua.

Wang Jian orcid: 0000-0002-7653-9577.
